# A cardiorespiratory classifier of voluntary and involuntary electrodermal activity

**DOI:** 10.1186/1475-925X-9-11

**Published:** 2010-02-25

**Authors:** Stefanie Blain, Sarah D Power, Ervin Sejdic, Alex Mihailidis, Tom Chau

**Affiliations:** 1 Institute of Biomaterials and Biomedical Engineering, University of Toronto, Toronto, ON, Canada; 2 Bloorview Kids Rehab, Toronto, ON, Canada; 3 Department of Occupational Therapy, University of Toronto, Toronto, ON, Canada

## Abstract

**Background:**

Electrodermal reactions (EDRs) can be attributed to many origins, including spontaneous fluctuations of electrodermal activity (EDA) and stimuli such as deep inspirations, voluntary mental activity and startling events. In fields that use EDA as a measure of psychophysiological state, the fact that EDRs may be elicited from many different stimuli is often ignored. This study attempts to classify observed EDRs as voluntary (i.e., generated from intentional respiratory or mental activity) or involuntary (i.e., generated from startling events or spontaneous electrodermal fluctuations).

**Methods:**

Eight able-bodied participants were subjected to conditions that would cause a change in EDA: music imagery, startling noises, and deep inspirations. A user-centered cardiorespiratory classifier consisting of 1) an EDR detector, 2) a respiratory filter and 3) a cardiorespiratory filter was developed to automatically detect a participant's EDRs and to classify the origin of their stimulation as voluntary or involuntary.

**Results:**

Detected EDRs were classified with a positive predictive value of 78%, a negative predictive value of 81% and an overall accuracy of 78%. Without the classifier, EDRs could only be correctly attributed as voluntary or involuntary with an accuracy of 50%.

**Conclusions:**

The proposed classifier may enable investigators to form more accurate interpretations of electrodermal activity as a measure of an individual's psychophysiological state.

## Background

Electrodermal activity (EDA) is one of the most popular methods of measuring arousal, attention and orientation in fields such as psychology [1], emotion recognition [2] and psychophysiology [3]. It consists of a slowly evolving baseline and quick, transient changes known as electrodermal reactions (EDRs), defined as increases in EDA of over 0.05 μS within five seconds [4]. EDRs are a result of cholinergenic stimulation of the sweat glands, causing increases in electrical conductance of the skin. These fluctuations in conductivity are interpreted as a measure of overall arousal of the sympathetic nervous system. While amplitude, latency and fall time are routinely reported, reporting the stimulus of an EDR remains challenging. In particular, it is difficult to discern among uncued increases in EDA due to: (1) spontaneous increase, often referred to as a non-specific EDR; (2) result of internal stimulation (e.g. mental stimulation, a large amplitude inspiration, biting the tongue); or (3) result of external stimulation (e.g. startling noises, changes in visual stimulation)[3,5-8].

Often, it is important to be able to attribute an EDR to one of the three aforementioned sources. For example; in the field of polygraphy, EDA is often measured as suspects are administered a series of questions, one of which pertains to knowledge of the details of the crime (i.e. the Guilty Knowledge Test or the Concealed Information Test). An EDR succeeding a crime-relevant question indicates that the suspect is lying, and can be used to detect 94.2% of innocent suspects and 83.9% of guilty suspects, under controlled conditions [9]. However, EDRs can be voluntarily generated using a variety of physical and mental activities, significantly decreasing the accuracy of the test. Clearly, having a method of distinguishing the involuntary guilty reaction from the voluntary mental activities would significantly enhance the reliability of polygraphy examinations. Differentiation between voluntary and involuntary electrodermal activity may also useful in the field of access pathways for individuals with severe and multiple disabilities. Numerous options have been explored to enable individuals without speech or reliable motor movement to interact with the environment, among them, the use of electrodermal activity as an access pathway [10,11]. While voluntarily generating EDRs to indicate intent remains a promising access pathway for these individuals, the use of this signal for precise and reliable communication has been contested on the grounds of high incidences of metabolic noise [12]; the ability to distinguish involuntary EDRs from voluntary ones would greatly enhance the robustness of this access pathway.

Despite enormous advancements in the procedures and equipment involved in recording electrodermal activity, Landis's [13] comments eighty years ago on the inability to attribute psychological significance to a single EDR are still applicable today. He provides an extensive list of EDR sources, illustrating the magnitude of the challenge of determining the origin of a single observed EDR. To address this challenge, many studies have been conducted in a controlled environment, enabling the assumption that all observed EDRs are a result of varying the stimulus of interest [6,14-16]. The validity of this assumption is often not discussed; in addition, many studies occur in environments that are not controlled. In these situations, Cacioppo and Tassinary [17] have suggested that in order to develop clear psychophysiological inferences of one signal, it is necessary to consider other physiological signals that may vary with the psychological event of interest. Studies that have examined patterns of physiological signals have clearly established that respiratory and cardiac artefacts also vary with many of the aforementioned EDR sources [18,19]. To date, numerous EDA studies have followed Cacioppo and Tassinary's suggestion, taking respiratory and cardiac signals into consideration in one of three ways:

1) Cardiac and respiratory signals are recorded simultaneously with electrodermal activity. However, all signals are analyzed independently, not taking into account the interaction among the signals [1,7,20,21].

2) The interaction among cardiac, respiratory and EDA signals is acknowledged. However, procedures for removing cardiac and respiratory artefacts are not described [22-24].

3) Cardiac, respiratory and EDA signals are recorded simultaneously. Features from each signal are extracted independently and used as independent inputs into a classifier that determines the overall source of all the EDRs recorded within the classification period [2,3,17,25-30].

The first two methods do not sufficiently account for the interaction between these physiological signals. The third method follows in the spirit of Cacioppo and Tassinary, improving classification accuracy by accounting for the changes in more than one physiological signal. However, while this third method is useful for classifying an individual's psychophysiological state based on a long term recording, it is unable to determine the source of a single EDR, a process necessary for the real-time application of polygraphy and access mentioned earlier. To date, few efforts have been made towards single EDR discrimination; Crone et. al [31] used the respiratory signal to eliminate heart rate and skin conductance changes associated with gross respiratory manoeuvres, and Schneider et. al [32] have developed a set of rule-based guidelines to eliminate respiration-related artefacts in EDA recordings.

While the aforementioned techniques exist to eliminate respiratory-related artifacts from the EDA signal, they typically involve manual, offline analysis of the respiratory signal, and are unsuited for real-time EDR classification. Additionally, there currently exists no means of distinguishing voluntarily generated EDRs from involuntary EDRs using respiratory information alone. The purpose of this study is to develop a classifier that uses information from non-EDA physiological signals, namely, respiration and heart rate, to classify the source of a single EDR into one of two categories: (1) a voluntarily generated EDR, including those generated by internal physiological processes such as inspiration and internal mental processes such as music imagery; and (2) an involuntary EDR, including those generated by external startling stimuli and non-specific EDRs.

## Methods

### Participants

A convenience sample of eight able-bodied individuals (3 males, mean age 26 ± 3 years) participated in this study. Participants did not have conditions that may have affected their physiological signals and/or their ability to perform the required tasks, including metabolic, cardiovascular, respiratory, psychiatric, or drug- or alcohol-related disorders. Participants also had normal, or corrected to normal, hearing, were electrodermally labile and had a periodic baseline respiration pattern. Ethical approval was received from the relevant institutions and all participants provided written consent.

### Instrumentation

**Figure 1:**
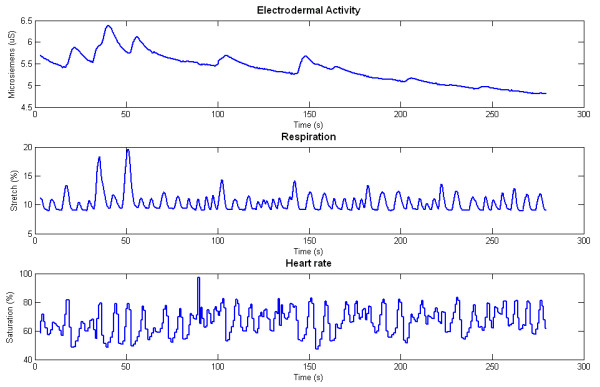
**Typical signals recorded from the Thought Technology equipment**. Raw electrodermal activity, respiration and heart rate signals recorded from the ProComp Infiniti hardware.

Three peripheral physiological signals were recorded from each subject using the ProComp Infiniti data acquisition system (Thought Technology). These were: (1) electrodermal activity, using two Ag/Ag-Cl gel-less electrodes attached to the medial phalange of the second and third fingers; (2) respiration, using a piezoelectric belt positioned around the subject's thoracic cavity; and (3) blood volume pulse, measured using an infrared sensor attached to the subject's fourth finger. All sensors were placed on the subject's non-dominant hand, and sampled at a frequency of 256 Hz. No additional filters or amplifiers other than those intrinsic to the ProComp Infiniti hardware were employed. Subjects were blindfolded and asked to don a pair of soundproof ear covers over a set of headphones, to ensure that the external stimuli being presented to each subject were fully controlled by the experimenter. Typical signals that were recorded from all sensors are presented in Figure 1.

### Protocol

Participants were seated comfortably in front of a computer as the sensors were attached. Prior to the data collection, participants were asked to choose several songs of their own preference and of the same valence (i.e. happy or sad), which they felt elicited a strong emotional reaction. The participants were informed that when cued in the experiment, they would be required to perform music imagery of one of their chosen songs, in other words, to sing the song vividly in their head. They were additionally informed that the purpose of the imagery was to elicit an emotional reaction, thus, when they began to feel emotionally habituated to their current song, they were requested to switch to another song. Visual inspection of the recorded physiological signals confirmed that music elicited sympathetic excitation in all participants. After choosing their songs, the participants performed the four sets of trials outlined in Table 1. The order of the trial presentation was randomized for each participant, and the participants performed the activities over two separate days to ensure maximum concentration and focus during each trial.

**Table 1:** Summary of Experimental Trials

Trial Block	Trial Description	Total Time	Time of Presentation of Startles (s)	Trials without noise	Trials with noise	Total trials
A	Quiet resting	2 min, 10 s	N/A	2	2	4

B	Music imagery	3 min, 40 s	N/A	2	2	4

C	Quiet resting with startles	2 min, 10 s	20, 45, 65, 90, 110	2	2	4

D	Music imagery with startles	3 min, 40 s	1) 31, 88, 111, 149, 191 2) 28, 82, 91, 128, 151 3) 10, 31, 72, 150, 189 4) 14, 89, 111, 170, 190	2	2	4

In Block A trials (quiet resting trials), subjects were instructed to relax and clear their minds of thought, keeping their bodies as still as possible. During Block B trials (music imagery), the investigators cued subjects every 20 seconds via a gentle tap on their arm to alternate between quiet resting and performing music imagery. For Block C trials (quiet resting with startles), participants received the same set of instructions as in Block A. At the time points indicated in Table 1, participants were presented with one of five auditory startling stimuli through their headphones. Characteristics of these stimuli are presented in Table 2. During Block D trials (music imagery with startles), participants received the same instructions as Block B trials, and one of the five auditory stimuli in Table 2 were presented at the time points indicated in Table 1. Prior to Block A and B trials, participants were asked to take 3 deep breaths over the course of 1 minute so as to elicit inspiratory-induced EDRs. Trials in all four blocks were conducted under two conditions: (1) in silence, and (2) in the presence of a continuous background noise (an air conditioner), yielding a total of 16 recorded trials. The presence of background noise has been noted to enhance startle reactivity in humans [33]; this condition was included to develop a classifier trained on EDRs generated in both controlled and naturalistic environments.

**Table 2:** Auditory Startle Sound Characteristics

Sound	Intensity (dB)
Dog bark	80 ± 2

Glass shattering	91 ± 2

Door slam	83 ± 3

Cough	79 ± 1

Sneeze	82 ± 1

### Proposed Cardiorespiratory Classifier

**Figure 2:**
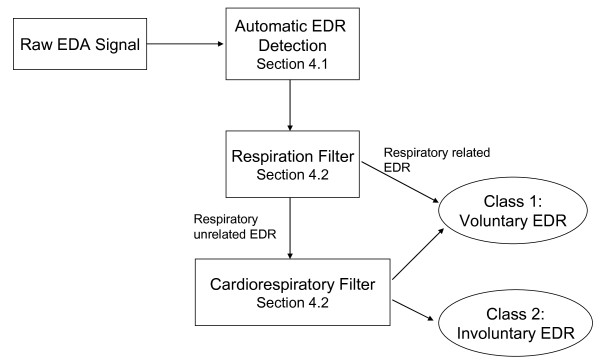
**Overview of the cardiorespiratory classifier**. Electrodermal reactions are identified from the raw EDA signal by the automatic EDR detector. These EDRs are subsequently tested by the respiratory and cardiorespiratory filters to determine whether they were voluntarily or involuntarily generated by the participant.

The following section will present the three elements that constitute the proposed cardiorespiratory classifier of electrodermal activity (Figure 2).

### Automatic EDR detection

**Figure 3:**
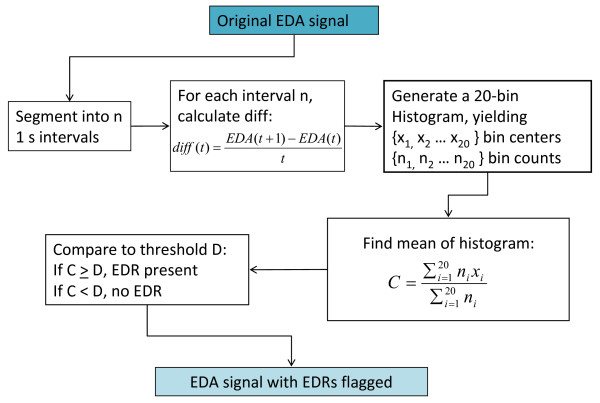
**Automatic EDR detection algorithm**. The mean of the histogram of the derivative of the EDA signal (C) is compared to the threshold (D) to determine whether a one second interval of EDA contains an EDR [10].

To detect EDRs, we employed the rule-based classifier proposed by Blain et al. [10]. Here, we only review the main concepts of the method and refer the reader to the original article for additional details. The gradient of baseline electrodermal activity is predominantly negative; during the initiation of an electrodermal reaction, this gradient becomes sharply positive. As a result, the first difference of the EDA signal is a discriminatory feature that indicates the presence of electrodermal reactions. In particular, the mean (C) of the distribution of the first difference of the EDA signal over a one second window can be used to detect the presence of an EDR [10]; this process is summarized in Figure 3.

The threshold (D), as referred to in Figure 3, must be determined for each individual experimental protocol, and is defined such that:

If C < D, the EDA signal from (t_i_, t_i _+ 1) contains no EDRs.

If C ≥ D, the EDA signal from (t_i_, t_i _+ 1) is part of an EDR.

D was chosen via an receiver operating characteristic analysis to simultaneously maximize sensitivity and specificity of EDR detection. To this end, a typical EDA signal was selected at random from a Block A trial of one of the participants. In this signal, five EDRs of varying amplitudes were identified manually. A maximum sensitivity and specificity of 100% were achieved at a value of D = 4 x 10^-4^. Using this threshold, a sensitivity and specificity of 100% were achieved for all trials of each of the eight subjects. This method of identifying EDRs is similar in principle to other methods that use first derivatives to define the start point, peak and end point of an observed EDR, but is more general in its abilities [34]. While it has the same ability to identify EDRs as the algorithm proposed by Frantzidis et al., this method does not have the ability to define the characteristics of the response.

### Respiration Filter

Having presented a method to detect EDRs, we now introduce a respiratory filter whose purpose is to remove respiration-induced EDRs. Deep inspirations are known sympathetic stimuli - subjects are often asked to take a deep breath while EDA equipment is being set up and calibrated, as it is an established method of generating an EDR [6,18]. The characteristics of the respiration signal as recorded via a piezoelectric belt have a large variance not only between subjects, but within subjects as well. In addition to natural circadian variations of respiratory patterns, the position and tension of the belt is not identical between trials, resulting in a large intrasubject variability. As a result, specific features cannot be used to classify a deep inspiration from typical respiration patterns. Instead, we propose to detect the point at which respiration patterns deviate significantly from a baseline respiration model developed for each session, for each participant. Details of our algorithm follow below.

The algorithm uses the respiration length line (RLL) to characterize each second of the respiration signal. RLL combines the measures of respiration rate and amplitude, and is a common measure of respiration suppression [3]. A decreased respiration rate and a decrease in respiration amplitude result in a shorter length line. Let the respiration signal generated by the expansion and contraction of the lung cavity be represented by *r(t) *and the sampling frequency of the signal be represented by *f *(in this protocol, *f *= 256 Hz). The respiration length line is produced by summing the Euclidean distance between successive points within a five second window of r(t), as presented in equation (1).(1)

This single measure of RLL is disproportionately affected by the starting point of measurement on the curvilinear respiration pattern. Following the solution outlined by Ben-Shakhar et al. [3] we address this problem by recalculating the RLL within a five second window 10 more times, each time beginning the measurement 0.1 seconds after the previous calculation. The average of these 10 measurements yields RLL_avg_(t) for each second of the recorded signal, as illustrated in equation (2).(2)

The 5% trimmed mean (μ_trim_) and trimmed standard deviation (σ_trim_) for the resultant RLL_avg_(t) signal are then calculated for the baseline signals, yielding robust measures of the distribution of respiration length lines during quiet breathing [35]. We define the respiratory threshold *ψ *as *ψ *= *μ*_*trim *_± 3*σ_*trim*_. For the remainder of the trials, each RLL_avg_(t) is compared against this respiratory threshold, such that if |*RLL*_*avg*_(*t*)| > *ψ*, the respiration signal contained in the 5 second window beginning at time t contains an irregular breath, i.e., one that departs from baseline respiration.

### The Bootstrap Variability Cardiorespiratory Classifier

#### Cardiorespiratory cross-correlation

Having screened out respiration-induced EDRs, we now present a filter to classify the remaining EDRs as voluntary (i.e., due to music imagery) or involuntary (i.e., due to auditory startle or spontaneous EDA fluctuations). The classification of EDRs into voluntary or involuntary responses requires several assumptions. The source of some EDRs, such as those generated by a deep inspiration, can be verified from the record of other physiological signals. However, in most other situations, the source of the EDR is unknown, and we must classify the EDRs based on the assumption that the participant is fully compliant, engaging in the specified mental task. Thus, in this study, all EDRs generated during periods of rest were assumed to be involuntary, and all electrodermal reactions generated during periods of music imagery were assumed to be voluntary. The presented cardiorespiratory filter tracks respiratory sinus arrhythmia (RSA), a phenomenon whose physiological origins are still debated wherein heart rate fluctuations at respiratory frequencies are observed in healthy humans [36,37]. The filter is based on the premise that we will observe a momentary lapse in the RSA of an individual during the generation of voluntary EDRs; in other words, voluntarily generated EDRs will be accompanied by a marked decorrelation between heart rate and respiration. The proposed method creates a statistical model of the expected correlation of the heart rate and respiration data while the individual is at rest, and using bootstrap prediction bands, determines whether a significant decorrelation between the two signals has occurred. This decorrelation is attributed to non-respiratory influences including the imagery and startle responses of the participants.

Let *R(t)*, 0 ≤ *t *≤ *T *represent the raw respiration signal, where T is the duration of the signal in seconds. Instantaneous heart rate, *HR*(*t*), in beats per minute (bpm), was computed by inverting the interbeat intervals extracted from the raw blood volume pulse (BVP) signal. The first derivative of the respiration signal *R*^'^(*t*) was estimated by the first difference of the sampled version of R(t). The relative heart rate changes, *HR*^'^(*t*), were calculated as follows,(3)

where δ is defined as in equation 1. Both *HR*^'^(*t*) and *R*^'^(*t*) were standardized to 0 mean and unit variance, yielding in *HR*^*z*^(*t*) and *R*^*z*^(*t*), respectively.

The two second cross-correlation in the *m*^*th *^segment,*C*_*m*_(*t*), between *HR*_*z*_(*t*) and *R*_*z*_(*t*) was calculated as(4)

where *m *- 1 ≤ *t *≤ *m *+ 1, *m *= 1,2,...,*M *and *M *= ⌈ *T *- δ ⌉ - 1 with ⌈ ⌉ denoting the ceiling function [38]. In other words, the cross-correlation between *HR*_*z*_(*t*) and *R*_*z*_(*t*) is calculated within a two-second sliding window with 50% overlap between successive windows. In the above, *C*_1_(*t*) is the cross-correlation between *HR*_*z*_(*t*) and *R*_*z*_(*t*), from 0 to 2 seconds; *C*_2_(*t*) is the cross-correlation between the same two signals from 1 to 3 seconds, and so on. Note that *HR*_*z*_(*t*) *R*_*z*_(*t*) signals change over similar timescales so that their cross-correlation is meaningful.

Therefore, for a signal of duration *T *seconds, we will have *T *two second cross-correlation curves. These *T *curves generated from the resting trial data were assumed to represent the typical correlation between heart rate and respiration in the absence of both environmental and internal stimuli. Following the formulation of Lenhoff *et al. *[*39*], we use these resting trial correlation curves to generate a resting model for the cardiorespiratory correlation, and use prediction bands to determine whether or not a test correlation curve belongs to the same population from which the resting curves were generated. If the test curve falls within the prediction bands, i.e., belongs to the population of resting trials, we conclude that the individual was in a resting state; if the test correlation curve falls outside of the prediction band, we conclude that the individual was affected by an internal or external stimulus. The cross-correlation curves are low harmonic curves, consequently, this method is reliable using as few as 25 curves [39]; the authors recommend a minimum of *T *= 30 seconds to generate a valid population model. The generation of the population model and the prediction bands is detailed below.

#### Generation of resting correlation curve model

The *T *curves generated from the resting trials are viewed as perturbations of a true curve that can be represented by the finite Fourier sum:(5)

where K is 512, and 0 ≤ t ≤ T. In equation (6), μ is a constant that represents the overall mean of the cardiorespiratory correlation curve, and the form of *f*(*t*) stipulates that *f*(0) = *f*(*T*) = μ. For each correlation curve, *C*_*m*_(*t*), we compute its Fourier representation *Ĉ*_*m*_(*t*) as follows(6)

where α_m,k _and β_m,k _are the coefficients for the Fourier approximation of the m^th ^curve. For each *Ĉ*_*m*_(*t*), we gather these fitted coefficients into a vector W_m _of length 2K + 1:(7)

The means of each of the coefficients in the T instances of W_m _(1 ≤ m ≤ T) were calculated and gathered into a 1 × (2K + 1) vector denoted ,(8)

and we also define vector ℓ(*t*) of length 2K + 1,(9)

From these two vectors, the sample mean curve M(t) can be estimated as(10)

where the superscript *t *denotes the transpose. The variability of M(t) is represented by S(t) and defined as,(11)

The mean and variability curves, i.e., M(t) and S(t), define the resting curve model for the participant.

#### Generation of prediction bands

Now that we have a resting cardiorespiratory correlation curve model, we need to define its boundaries of membership. In other words, when do we consider a correlation curve as belonging to the resting model? One way to define this membership is to construct prediction bands around the mean curve [39-41], such that curves lying within the prediction bands are considered as belonging to or arising from the resting model.

The following procedure is used to generate prediction bands. As above, suppose that we have *M *cross-correlation curves between resting heart rate and respiration signals. We randomly select a bootstrap sample of *M *- 1 curves, with replacement, from this population of *M *resting correlation curves. This is repeated N_B _times, where *N*_*B *_> > 1. For the *i*_*th *_bootstrap sample, *i *= 1,...,*N*_*B*_, we calculate the mean and variability curves, *M*_*i*_(*t*) and *S*_*i*_(*t*), as in equations (10) and (11). For each bootstrap sample, let *Ĉ*_*j*_(*t*) represent the Fourier approximation to the cross-correlation between the *j*^*th *^respiration and heart rate signals, *j *= 1,...,*M *- 1. We then calculate the standardized difference, *D*_*ij*_(*t*), between the *j*^*th *^curve, *Ĉ*_*j*_(*t*), from the *i*^*th *^bootstrap sample and the mean curve of the same bootstrap sample, *M*_*i*_(*t*),(12)

For the *i*^*th *^bootstrap sample, *i *= 1,...,*N*_*B*_, we obtain *D*_i_* as the maximum difference over all *M *- 1 curves, over time.(13)

Given a desired confidence level 100(1-α)%, we chose the constant θ, so that(14)

where α is 0.05 in the present study. In other words, θ is chosen such that there is a 95% probability that the maximum standardized difference between any curve and the mean curve is less than or equal to θ. Finally, the upper and lower 95% prediction bands were calculated as(15)

where *M*(*t*), *S*(*t*) and θ are given by equations (10), (11) and (14), respectively. Any correlation curve bounded by *U*(*t*) and *L*(*t*) is considered as arising from the resting curve model given by *M*(*t*) and *S*(*t*). A resting model and the associated prediction bands were estimated individually for each participant.

#### Testing the membership of unknown cardiorespiratory data

For a given participant, each two seconds of cardiorespiratory data from the non-resting trials (blocks B, C, and D) were tested against the prediction bands of the resting model, thereby determining whether or not these data resembled the resting cardiorespiratory correlation curves. In essence, we are thus determining whether or not external influences are mediating cardiac activity.

For each *T *seconds of data, where *T *represents the total length of the trial in seconds, we calculate *Q*(*s*), 0 ≤ *t *≤ *T *and *t *- 1 ≤ s ≤ *t *+ 1, which is the Fourier approximation to the cross-correlation between a two second segment of *HR*_*z*_(*t*) and the corresponding two second segment of *R*_*z*_(*t*) from a non-resting trial. We then calculate the standardized difference, *D*(*s*), between the unclassified correlation curve and the resting mean curve,(16)

where *M*_*R *_and *S*_*R *_are the mean curve and the standard deviation curves estimated from the resting trials. If the maximum absolute standardized difference from the resting mean,  is less than or equal to θ, that is, the unclassified correlation curve is bounded by the upper and lower prediction bands, then the test segment of data is classified as resting state. Otherwise, the test segment is considered as being influenced by external processes. For convenience, we created an indicator pulse spanning the duration of the test signal,(17)

where, as before, 0 ≤ *t *≤ *T *and *t *- 1 ≤ *s *≤ *t *+ 1. This indicator function is used in Section 4.4 to determine the source of a single, observed EDR.

### Classifier Evaluation

All detected EDRs were subsequently validated by visual inspection. In addition, each electrodermal activity signal was visually inspected for undetected EDRs. Here, an EDR was defined as an increase in the EDA signal of over 0.02 μS within five seconds. When the automatic detection algorithm flagged an EDR, the heart rate and respiratory signals were segmented beginning two seconds preceding the onset of EDR detection and ending one second following the onset of EDR detection. This window was chosen to account for the difference between the latency of the heart rate response (0.25 to 2 seconds) [42], and the latency of an electrodermal response (1.3 to 2.5 seconds) [43]. Segmentation thus yielded a 3 second segment for analysis by the cardiorespiratory classifier described in Section 4.3, generating a corresponding indicator function *I*(*t*). If *I*(*t*) = 1 (i.e., significantly different from the resting model) at any time within the segmented signal, the detected EDR was classified as voluntary, otherwise, it was classified as involuntary. For each subject, the number of true positives (TP), i.e., correctly classified voluntary reactions, including EDRs generated by a deep inspiration; true negatives (TN), i.e., correctly classified involuntary reactions, including EDRs generated from a startling stimulus; false positives (FP), i.e., incorrectly classified voluntary reactions; and false negatives (FN), i.e., incorrectly classified involuntary reactions were recorded. From these values, the positive predictive value (PPV), negative predictive value (NPV) and overall accuracy of the classifier were calculated.

Classification results were compared for EDRs generated during trials conducted in silence and trials conducted in the presence of a background noise. These two conditions were compared with a Pearson's chi-squared test (df = 1) to determine whether classification accuracy differed significantly between trials conducted in the presence and absence of background noise.

## Results

### Automatic EDR Detection

In all the signals across all subjects, 100% of the EDRs present in the signals were detected, and no false positives were generated. The number of detected EDRs varied between participants; these results are presented in Table 3.

**Table 3:** Individual Cardiorespiratory Classifier Parameters

Subject	Number of Detected EDRs	Respiratory Threshold (ψ)		Cardiorespiratory Threshold (θ)
			
		Lower	Upper	
1	74	0.004	0.01	0.2634

2	80	0.004	0.009	0.1766

3	111	0.004	0.008	0.1988

4	57	0.003	0.014	0.2523

5	88	0.003	0.009	0.1855

6	31	0.003	0.007	0.2252

7	100	0.003	0.005	0.2451

8	33	0.003	0.018	0.1700

### Respiratory and Cardiorespiratory Filter Parameters

The respiratory and cardiorespiratory patterns varied significantly between participants. As a result, the threshold values ψ for the respiratory filter and θ for the cardiorespiratory filter were unique to each individual; these values are listed in Table 3.

### Classification Results

PPV and NPV for each participant were calculated according to the truth set defined by the rules presented in Section 4.4. These results along with the overall accuracy of single EDR classification are presented for each participant in Table 4.

**Table 4:** Cardiorespiratory filter classification results

Participant	PPV	NPV	Accuracy
1	77%	83%	80%

2	79%	82%	80%

3	82%	74%	78%

4	71%	73%	72%

5	69%	71%	72%

6	94%	92%	90%

7	82%	86%	83%

8	67%	83%	70%

**Average**	**78 ± 9%**	**81 ± 7%**	**78 ± 7%**

**Figure 4:**
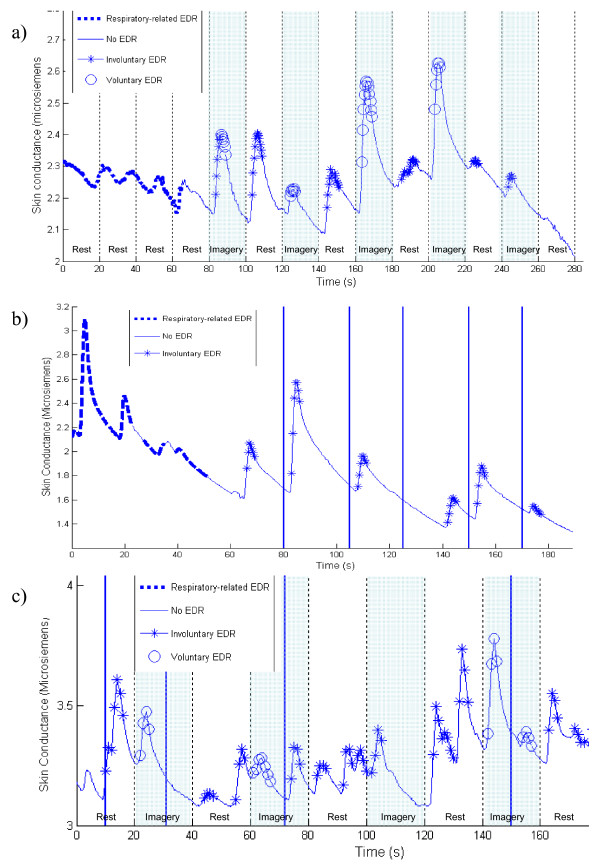
**Classification of EDRs**. Classification of EDRs within: a) an imagery trial (Block B); b) a quiet resting trial with startles (Block C); and c) an imagery with startles trial (Block D). Solid vertical lines denote the times at which audio startles were presented.

Examples of classified trials are presented in Figure 4. Figure 4a presents a trial wherein the individual alternated between 20 second periods of rest and activity. In this trial, each detected EDR was correctly classified with the exception of that generated in the final imagery period. Figure 4b presents a baseline trial during which startling noises are presented; four of the five audio stimuli produced a startle EDR, all of which were correctly classified as involuntary reactions. The classifier also correctly identified the two spontaneous EDRs in this trial as involuntary. In Figure 4c, the participant alternated between rest and music imagery while audio stimuli were presented at random intervals; two of the audio stimuli (at 10s and 72s) generated EDRs, which were correctly classified as involuntary. Voluntary EDRs were correctly classified in all imagery periods with the exception of the EDR at 100s. All involuntary EDRs were also correctly identified.

### Effect of Presence of Background Noise

Trials conducted in silence were compared to trials conducted in the presence of low-level background noise for each participant. Table 5 illustrates that for all except one participant, there was no significant difference in classification accuracy. However, for partcipant 2, EDRs generated during trials conducted without background noise were more accurately classified (p = 0.02) than those generated in the presence of background noise.

**Table 5:** Accuracy of classifying EDRs generated with and without background noise

Participant	Without background noise (total # EDRs)	With background noise (total # EDRs)	p
1	78.3% (60)	92.9% (14)	0.517

2	85.7% (49)	71.0% (31)	0.02

3	78.9% (71)	77.5% (31)	0.84

4	69.4% (49)	87.5% (8)	0.27

5	71.0% (69)	73.7% (19)	0.81

6	91.3% (23)	87.5% (8)	0.61

7	78.7% (47)	80.7% (57)	0.25

8	70.0% (20)	69.2% (13)	0.95

## Discussion

This study proposes a method of classifying single EDRs as voluntary or involuntary by utilizing cardiorespiratory signals that are recorded simultaneous with electrodermal activity. Distinguishing between resting and active states without the help of the classifier would require the assumption that all observed EDRs were generated due to mental imagery. In this situation, classification of the EDA signal would decrease in accuracy from 79 ± 7% to 50 ± 8%, demonstrating that a cardiorespiratory classifier based on the respiration length line and the cross-correlation of heart rate and respiration significantly improves the ability to determine the source of an observed EDR.

### Classification Assumptions

The participants recruited to this study were not trained in mental control techniques, such as meditation. Therefore, it is likely that at some point during the periods of rest, the participants' minds were not entirely cleared, and a mentally stimulating thought caused an EDR. This EDR would be preceded by a decorrelation between the respiratory and cardiovascular signals, as it was voluntarily generated by the mental stimulus. However, as it occurred during the resting period, this EDR would be considered misclassified under the assumption that all EDRs generated during a resting period were involuntary. The converse situation may also occur; spontaneous EDRs are generated 7.5 times every minute in the average population [5]; it is likely that during an imagery period, the participant experienced a spontaneous EDR that was not preceded by a decorrelation between respiratory and cardiovascular patterns. The presented classifier would correctly label this reaction as involuntary, yet under the study assumptions, this classification would be erroneous, as the EDR occurred during an imagery period. While the authors recognize this problematic situation, data have not been gathered to provide any further information on the true source of the electrodermal reaction. Consequently, the assumption of full compliance to the required mental task is necessary, though as a result, the accuracy of the presented classifier is likely underestimated.

One recurring situation highlights the potential classification errors due to this assumption. Often, when cued to switch from a period of music imagery to a period of rest (ex. during experimental blocks B and D), an electrodermal reaction is generated within the first five seconds of the rest period, and is classified as a voluntary reaction. As it occurs during a rest period, this EDR is considered to be a false positive. However, many participants reported that it was difficult to stop the music imagery process on cue, and that the act of ceasing to perform music imagery required more effort than initiating music imagery. This effortful act of abruptly terminating music imagery may well result in a voluntary EDR as the resting process is initiated. Taking this into consideration, if EDRs that occur within the first five seconds of an imagery to rest transition are considered voluntary, the positive predictive value significantly increases from 77% ± 8.7% to 87% ± 8.7% (p = 0.04), illustrating that the current reported classification accuracy is likely an underestimation of the true performance of the classifier.

### Effects of Background Noise and Time

In their 1963 study on animal startle reactions, Hoffman and Flesher serendipitously discovered that the background noise they were using to mask unpredictable environmental sounds in fact had an enhancing effect on startle reactivity in the rat [44]. This result has been replicated many times, and recently, the same phenomenon of increased startle reactivity during increased background noise has been demonstrated in humans [33]. In the context of these previous studies, it is intriguing that the results indicate that for seven of the eight participants, the classifier performs equally well for startle EDR generated under both conditions. This can potentially be explained with Holand's finding that a component of the overall startle response included an increase in blood pressure and heart rate [45]. The results from this present study suggest that while the magnitude of the startle reaction may be enhanced in the presence of background noise, the classifier remains robust against these changes, and is able to perform equally well under both conditions. In the case of participant 2, the classifier performed significantly better under conditions of silence. This difference may be attributed to either: a) distraction from the music imagery task in the presence of background noise or; b) a different pattern of cardiovascular startle response in the presence of background noise for this particular individual. Further investigation of this participant's responses to determine the source of the preferential classification is warranted.

### Limitations

While the ability for the classifier to distinguish between voluntarily and involuntarily generated EDRs appears promising, the results must be interpreted in light of the limitations of the study design. The classifier was tested on eight, able-bodied individuals within a narrow age range, who may not have demonstrate significant differences in their patterns of electrodermal activity. This is illustrated in the fact that parameter D, the threshold for detecting an EDR, which was determined from one randomly chosen subject was 100% suitable for the remaining 7 subjects. Electrodermal activity has been known to vary with age, and among individuals with different disabilities. Further studies are needed to determine suitable parameter values for EDR detection in individuals outside the demographics of those who participated in this study. Furthermore, all physiological signals were recorded under controlled environmental conditions (minimal radio frequency interference). The values of the parameters presented in this study are thus specific to uncontaminated physiological signals. Application of the proposed classifier amid noisy experimental conditions would require specific removal of the offending artefacts.

### Significance of Study

In fields where electrodermal activity is used as a measure of the state of an individual's sympathetic nervous system, some means of artifact control must be employed to determine the source of the EDRs (i.e. whether they are voluntarily or involuntarily generated). Until now, these methods have not existed, with the exception of a recently-developed standardized rule-base that utilizes visual inspection of a respiratory signal to determine whether or not an EDR is a respiratory artefact [32]. While useful for identifying EDRs that were generated from changes in respiration, these rules do not distinguish voluntary from involuntary EDRs. As there is no existing means of making this distinction, every discipline makes different generalizing assumptions about the source of the observed EDRs. In the field of polygraphy, all electrodermal reactions are assumed to be involuntary, and indicative of the subject's unconscious reactions, despite evidence illustrating that mental exercises are an effective countermeasure and can be used to voluntarily generate EDRs to bias the results [3]. In the field of access technologies, all electrodermal reactions generated are assumed to be voluntary, in spite of a priori knowledge that spontaneous EDRs occur at an average rate of 7.5 per minute [5]. Fields of study that include electrodermal activity as a measure of sympathetic arousal may benefit from using the proposed classifier to obtain greater insight into the source of observed EDRs, provided that the relevant cardiorespiratory signals can be simultaneously obtained.

## Conclusions

This paper has proposed a method for classifying EDRs using simultaneously recorded cardiac and respiratory signals. The presented classifier tracked both the RLL over a five second moving window, and the cross-correlation between the respiratory and heart rate signals, to distinguish voluntary EDRs due to an irregular breath or mental imagery, from involuntary EDRs associated with startle reactions or a spontaneous increases in EDA. This classifier had a positive predictivity of 78%, a negative predictivity of 81%, an overall accuracy of 79%, and, with the exception of one subject, performed equally well under conditions of silence and background noise. This is nearly a 30% improvement in accuracy over the case when all EDRs are naively assumed to be voluntarily generated. Our results suggest that the cardiorespiratory classifier may be useful for EDA research, such as polygraphy or alternative access for individuals with disabilities, where the source of single EDRs is of particular interest.

## Competing interests

The authors declare that they have no competing interests.

## Authors' contributions

SB carried out the study, developed and tested the classification algorithm, and performed the statistical analysis. SP designed and carried out the data collection sessions. ES helped to draft the final manuscript. AM participated in project direction, funding and manuscript editing. TC conceived of the study, directed the statistical analysis, participated in algorithm development and managed the project. All authors read and approved the final manuscript.
